# A Database of Plastid Protein Families from Red Algae and Apicomplexa and Expression Regulation of the *moeB* Gene

**DOI:** 10.1155/2015/510598

**Published:** 2015-05-31

**Authors:** Oleg A. Zverkov, Alexandr V. Seliverstov, Vassily A. Lyubetsky

**Affiliations:** Institute for Information Transmission Problems of the Russian Academy of Sciences (Kharkevich Institute), Bolshoy Karetny Pereulok 19, Moscow 127994, Russia

## Abstract

We report the database of plastid protein families from red algae, secondary and tertiary rhodophyte-derived plastids, and Apicomplexa constructed with the novel method to infer orthology. The families contain proteins with maximal sequence similarity and minimal paralogous content. The database contains 6509 protein entries, 513 families and 278 nonsingletons (from which 230 are paralog-free, and among the remaining 48, 46 contain at maximum two proteins per species, and 2 contain at maximum three proteins per species). The method is compared with other approaches. Expression regulation of the *moeB* gene is studied using this database and the model of RNA polymerase competition. An analogous database obtained for green algae and their symbiotic descendants, and applications based on it are published earlier.

## 1. Introduction

The concept of orthology and construction of orthology databases are important areas of bioinformatic research. However, the orthology relationship is not yet decisively formalized and some of its important features may depend on taxonomic context of the data and properties of particular organelles. Mathematically, identification of orthologs corresponds to building clusters in a graph with its vertices assigned gene or protein sequences. The majority of clustering methods utilize various strategies to weight the graph edges with subsequent construction of “highly connected components,” that is, clusters resulting from a certain clustering procedure.

The edge weight reflects similarity of amino acid sequences generated in various pairwise alignment procedures, intron content and positioning, protein domain architecture, gene synteny, and so forth. Usually the weights are computed with global alignment using the Needleman-Wunsch algorithm, or local alignment using BLAST. Various clustering approaches were proposed, from specifically organized partitioning of the spanning tree of the initial graph (the originally proposed algorithm ClusterZSL, refer to [1]) to time estimation of random walk on a graph (the OrthoMCL algorithm). In the latter algorithm based on Markov clustering, walk within a cluster is long, and jumps between clusters are rare [2]. Due to heuristic nature of these processes, comparison of the algorithms cannot be formalized, especially in the absence of standard benchmarking data. The description of OrthoMCL implicitly states that its convergence is difficult to discuss even in hypothesis.

The algorithm ClusterZSL essentially differs from commonly employed methods, including OrthoMCL, by not using the mutual-best-hit criterion. For a pair of genomes, a gene may produce none or many best hits; the latter is especially the case when considering suboptimal hits that may in fact represent true orthologs. In contrast with other methods, ClusterZSL also minimizes the amount of paralogs in each cluster that in general seems a reasonable property. ClusterZSL can consider gene positioning in DNA and orthologous context of the gene neighborhood. A version of this algorithm that uses gene synteny was applied to various chordate animals and will be described in a separate publication.

The algorithm ClusterZSL and its computer program implementation possess the computational complexity of maximum *n* ^2^ accurate to a coefficient. The OrthoMCL uses matrix multiplication, the operation with the minimal complexity *n* ^*ω*^, where the exponent *ω* is a parameter. For the Gauss algorithm *ω* = 3 and for the Strassen algorithm *ω* = log_2_⁡7 ≈ 2.81 [3]. An asymptotically faster algorithm is known, which, however, takes advantage only with matrices of very high order and is practically of little use [4]; also refer to [5, 6]. Further concerns with the OrthoMCL algorithm are the estimation of the number of iterations (including matrix multiplications) and proof of convergence. The convergence requirement is obviously met with ClusterZSL. The running time of OrthoMCL appears to be much longer than that of ClusterZSL, at least with our testing data. Due to high scalability, performance of ClusterZSL does not depend on the amount of CPUs, which is a valuable practical property; the authors are unaware of attempts to assess the scalability of OrthoMCL.

Compare ClusterZSL with the algorithm used in the Ensembl database. Both start from the spanning tree. On later stages, the Ensembl algorithm relies in many respects on multiple alignments of leaf proteins, the task exponential in computational complexity if the alignment is optimized [7]. For alignment construction, the algorithm integrates the *M*-Coffee algorithm [8] or Mafft for larger data [9]. Both mentioned alignment procedures are heuristic and do not guarantee global minimization of the used functional. The ClusterZSL algorithm does not utilize multiple alignment.

Worth mentioning is another clustering method to establish orthology that was previously used by the authors. When the size of the clusters is known, for example, in studies of multicomponent systems where the length of the orthologous series is known for one component, the most dense cluster of the known size is constructed using the algorithm described in [10, 11]. We do not compare with phylogenetic methods here; for instance, refer to [12]. Note that the phylogenetic position of a species or protein belonging to any species is not always known.

The problem of the transcription factor regulon definition is of great interest. In red algae, the only plastid-encoded transcription factors are Ycf27, Ycf28, Ycf29, and RbcR (Ycf30). Of little information on them, the RbcR binding sites are known to vary even among close species [13], which hampers their detection. We will consider this problem on the example of the factor Ycf28, which, as it turned out, regulates the expression of the gene* moeB*.

In this study, the gene* moeB*, which is itself an important object of research, is tackled in a case study of gene expression regulation using ClusterZSL. This gene encodes an E1-like family enzyme involved in molybdopterin and thiamine biosynthesis. This family includes proteins that catalyze the adenylation by ATP of the carboxyl group of the C-terminal glycine in sulfur carrier proteins, for example, MoaD or ThiS. Bacterial proteins with domains characteristic for this family are described in [14]. The* moeB* gene is present in plastids of all sequenced Rhodophyta; refer to Table 1. Its ortholog in* Porphyra purpurea* and* Pyropia* spp. is* ORF382*, in* Cyanidium caldarium chlN*. In* P. perforata* the neighboring genes* moeB* and* ORF382* encode the N- and C-termini of the MoeB protein.

As evident from Table 1, the neighbor of* moeB* on the opposite strand is* trnW* that encodes the tryptophanyl-tRNA. In* Porphyra purpurea*,* Pyropia haitanensis*, and* Pyropia perforata* the genes* trnW *and* moeB* are separated by the short coding frame* ORF75*. The only exception is* Porphyridium purpureum*, where the neighborhood of* moeB* lacks a reliably highly transcribed gene on the opposite strand; refer to [15–23].

In this study we describe a database ClusterZSL of orthologous plastid proteins in red algae, secondary and tertiary rhodophyte-derived plastids, and Apicomplexa (the RedLine at May 2014 from the GenBank; also refer to http://lab6.iitp.ru/ppc/redline50/), constructed with the same algorithm ClusterZSL.

We use it in a case study of transcription regulation of the* moeB *gene. An analogous database obtained for green algae and their symbiotic descendants (the green line) and its applications are published in [1, 24–26].

Some recent papers ([27] et al.) glance upon plastid proteins the database CpBase, http://chloroplast.ocean.washington.edu/. It represents 35 plastomes from RedLine in comparison with 50 plastomes represented in the database ClusterZSL. The authors are not aware of the description of the method, which the CpBase has been constructed with, as well as the details related to it.

## 2. Materials and Methods

All plastid proteins are available in GenBank [28]. Orthology was established with the ClusterZSL algorithm described in [1] and applied previously in [24–26]. The algorithm parameters were set to *H* = 0.6, *L* = 0. Gene annotations were verified with the Pfam [29] and Prosite [30] databases.

Promoters were predicted using an algorithm described in [24, 31, 32]. For different *σ*-subunits of bacterial type RNA polymerases it utilizes data on mutation profiles of the* psbA* promoter in* Sinapis alba* [33] and other experimentally studied promoters [34].

In searches for motifs in the 5′-leader regions of* moeB* we used the original algorithm published in [35, 36] and the WEB service MEME [37], although the motifs were not detected.

The notion of the phylogenetic distribution (profile) is defined in [26]: for a given gene/protein *g*, it is a function on a given set *S* of species that equals (for all *s* from *S*) +1 if *g* is present in *s*, and −1 otherwise.

In Section 3 we essentially exploit the originally proposed model of RNA polymerase competition [38, 39]. The model describes the following situation. In DNA locus transcription many RNA polymerases involved simultaneously bind with the promoters of their type and elongate along their chains, possibly towards each other. This leads to the interaction of RNA polymerases, both between each other and with various protein and structural factors on DNA and RNA. As a result, the transcription levels of the genes significantly change, right up to inability to initiate the transcription of the divergent located gene (below in this role* moeB*), when an actively transcribed gene (resp.,* trnW*) plays against it, provided the intergenic region is not organized in a special way.

## 3. Results

We report the database ClusterZSL (http://lab6.iitp.ru/ppc/) of plastid protein families from red algae, secondary and tertiary rhodophyte-derived plastids, and Apicomplexa (the RedLine). The families contain proteins with maximal sequence similarity and minimal paralogous content and are built using the ClusterZSL algorithm. The database contains 6005 protein entries, 513 families, and 278 nonsingletons (from which 230 are paralog-free, and among the remaining 48, 46 contain at maximum two proteins per species and 2 at maximum three proteins per species). The comparison of the obtained protein families with the biological annotations indicates their good conformity.

**Figure 1:**
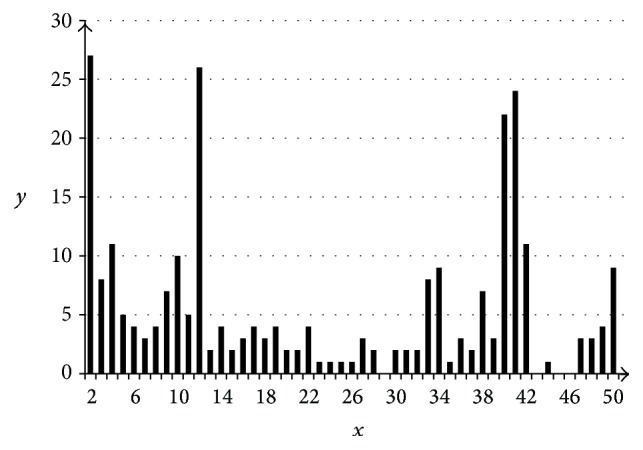
Distribution of cluster (*y*-axis, the ordinate) versus species (*x*-axis, the abscissa) numbers.

Tables 1 and 2 describe two clusters of the database.  Figure 1 presents a diagram of species content in inferred clusters.

Standard bacterial type promoters were not detected in the 5′-leader regions of* moeB*. However, the {A, T}-rich regions found upstream* moeB* may represent functioning −10 promoter boxes. Based on modeling RNA polymerases competition we suggest that the promoters of* moeB* are located in between* moeB *and* trnW* (refer to the Conclusions) and differ distinctly from the common template.

The presented database allows comparing a cluster of a gene (e.g.,* moeB*) with all other clusters. Phylogenetic distributions of* moeB* and Ycf28 coincide; for example, there is a unique transcription factor, which is encoded in a plastid if and only if* moeB* is encoded in it; it is Ycf28. That indicates that the best hit against* moeB* is Ycf28, a transcription factor.

The lack of detected −35 box for* moeB* naturally suggests that Ycf28 is an activator. Based on the same modeling, we surmise that the Ycf28 binding sites are located in between genes* moeB *and* trnW*. The only exception might be* Porphyridium purpureum*. The Ycf28 binding motif itself was not identified, probably due to the variability of binding sites.

Note that the 5′-UTRs of* moeB* are usually short and allow for very limited secondary RNA folding [40]. No conserved structures potentially regulating translation initiation were found that also suggests presence of transcription regulation.

## 4. Conclusions

The Ycf28 proteins are present in plastids of all Rhodophyta; refer to Table 2. In* Cyanidioschyzon merolae* and* Porphyridium purpureum* this protein is notably shorter.

In the presented database, phylogenetic distributions of* moeB* and transcription factor Ycf28 coincide. This observation leads to the suggestion that Ycf28 is a transcription regulation factor for* moeB*. The factor Ycf28 is a close homolog of the cyanobacterial transcription factor NtcA involved in regulation of nitrogen metabolism [41, 42]. Among cyanobacterial genes under the NtcA regulation only two have homologs in plastids. These are the genes of the factor itself and the regulatory protein GlnB from the family PII [43]. However, GlnB is rarely found in plastids, and the corresponding 5′-UTRs lack the conserved motif typically binding NtcA in cyanobacteria [41, 42]. This may suggest that the plastid-encoded Ycf28 and cyanobacterial NtcA are involved in different regulations.

In most species, presence of the actively transcribed tRNA gene* trnW* on the opposite strand precludes* moeB* transcription from a promoter located upstream that of* trnW* due to inevitable strong RNA polymerase competition. An important role of such competition in expression of closely located antidirected genes is substantiated in modelling and various experiments on gene expression. Such evidence includes data on bacterial type RNA polymerases *σ*-subunit knockout in plastids of* Arabidopsis thaliana* and data for mitochondrial RNA polymerases of the phage type [38, 39]. Therefore, the* moeB* promoter is likely to be located in between genes* moeB *and* trnW* and requires transcription initiation due to absence of an evident −35 box. Considering polymerase competition at these genes, the transcription factor binding site is likely to occur in the same region between the genes. Indeed, a binding site within an intensively transcribed region is unlikely effective due to interference of the factor with RNA polymerases.

Notably, short conserved motifs adjoining {A, T}-rich regions at their 3′-end are commonly found upstream* moeB*. This may be related to a low GC-content in plastids of most species. However, the predicted location of the binding site makes the putative mechanism of expression regulation specific to* moeB*.

## Figures and Tables

**Table 1:** Orthologs of *moeB* in plastids of rhodophyte algae as inferred with ClusterZSL and their genomic neighborhoods.

Class	Species	Locus	Protein MoeB	Genomic context
Bangiophyceae	*Porphyra purpurea *	NC_000925	NP_053945.1	(*trnW*)-*ORF75*-*moeB *
Bangiophyceae	*Porphyridium purpureum *	NC_023133	YP_008965710.1	(*ORF144*)*-ycf38*-*moeB *
Bangiophyceae	*Pyropia haitanensis *	NC_021189	YP_007947865.1	(*trnW*)-*ORF75*-*moeB *
Bangiophyceae	*Pyropia perforata *	NC_024050	YP_009027619.1	(*trnW*)-*ORF75*-*moeB *
Bangiophyceae	*Pyropia yezoensis *	NC_007932	YP_537017.1	(*trnW*)-*moeB *
Bangiophyceae	*Cyanidioschyzon merolae *	NC_004799	NP_849016.1	(*trnW*)-*moeB *
Bangiophyceae	*Cyanidium caldarium *	NC_001840	NP_045115.1	(*trnW*)-*moeB *
Florideophyceae	*Calliarthron tuberculosum *	NC_021075	YP_007878185.1	(*trnW*)-*moeB *
Florideophyceae	*Chondrus crispus *	NC_020795	YP_007627343.1	(*trnW*)-*moeB *
Florideophyceae	*Gracilaria salicornia *	NC_023785	YP_009019560.1	(*trnW*)-*moeB *
Florideophyceae	*Gracilaria tenuistipitata *	NC_006137	YP_063552.1	(*trnW*)-*moeB *
Florideophyceae	*Grateloupia taiwanensis *	NC_021618	YP_008144807.1	(*trnW*)-*moeB *

The *moeB* orthologs are also denoted by *moeB*, irrespective of corresponding original annotations. Genes on the opposite strand to *moeB* are given in brackets.

**Table 2:** Orthologs of Ycf28 in plastids of Rhodophyta as inferred with ClusterZSL.

Class	Species	Locus	Protein Ycf28	Bit score	*E*-value
Bangiophyceae	*Porphyra purpurea *	NC_000925	NP_053952.1	50.9	9.5*e* − 14
Bangiophyceae	*Porphyridium purpureum *	NC_023133	YP_008965713.1	48.6	5.0*e* − 13
Bangiophyceae	*Pyropia haitanensis *	NC_021189	YP_007947872.1	52.9	2.2*e* − 14
Bangiophyceae	*Pyropia perforata *	NC_024050	YP_009027626.1	53.3	1.7*e* − 14
Bangiophyceae	*Pyropia yezoensis *	NC_007932	YP_537023.1	55.2	4.4*e* − 15
Bangiophyceae	*Cyanidioschyzon merolae *	NC_004799	NP_849012.1	29.5	4.5*e* − 07
Bangiophyceae	*Cyanidium caldarium *	NC_001840	NP_045121.1	55.8	2.8*e* − 15
Florideophyceae	*Calliarthron tuberculosum *	NC_021075	YP_007878179.1	43.9	1.5*e* − 11
Florideophyceae	*Chondrus crispus *	NC_020795	YP_007627337.1	31.7	9.1*e* − 08
Florideophyceae	*Gracilaria salicornia *	NC_023785	YP_009019566.1	29.0	6.5*e* − 07
Florideophyceae	*Gracilaria tenuistipitata *	NC_006137	YP_063558.1	32.6	4.8*e* − 08
Florideophyceae	*Grateloupia taiwanensis *	NC_021618	YP_008144797.1	33.6	2.4*e* − 08

The last two columns contain estimates for the Pfam Crp-like helix-turn-helix domain (PF13545).

## References

[1] Lyubetsky V. A., Seliverstov A. V., Zverkov O. A. (2013). Elaboration of the homologous plastid-encoded protein families that separate paralogs in magnoliophytes. *Mathematical Biology and Bioinformatics*.

[2] van Dongen S., Abreu-Goodger C. (2012). Using MCL to extract clusters from networks. *Methods in Molecular Biology*.

[3] Strassen V. (1969). Gaussian elimination is not optimal. *Numerische Mathematik*.

[4] Coppersmith D., Winograd S. (1990). Matrix multiplication via arithmetic progressions. *Journal of Symbolic Computation*.

[5] Le Gall F. Powers of tensors and fast matrix multiplication.

[6] Smirnov A. V. (2013). The bilinear complexity and practical algorithms for matrix multiplication. *Computational Mathematics and Mathematical Physics*.

[7] Vilella A. J., Severin J., Ureta-Vidal A., Heng L., Durbin R., Birney E. (2009). EnsemblCompara GeneTrees: complete, duplication-aware phylogenetic trees in vertebrates. *Genome Research*.

[8] Wallace I. M., O'Sullivan O., Higgins D. G., Notredame C. (2006). M-Coffee: Combining multiple sequence alignment methods with T-Coffee. *Nucleic Acids Research*.

[9] Katoh K., Standley D. M. (2013). MAFFT multiple sequence alignment software version 7: improvements in performance and usability. *Molecular Biology and Evolution*.

[10] Galashov A. E., Kel’manov A. V. (2014). A 2-approximate algorithm to solve one problem of the family of disjoint vector subsets. *Automation and Remote Control*.

[11] Kel'manov A. V., Romanchenko S. M. (2014). FPTAS for solving a problem of search for a vector subset. *Diskretnyi Analiz i Issledovanie Operatsii*.

[12] Zmasek C. M., Eddy S. R. (2002). RIO: analyzing proteomes by automated phylogenomics using resampled inference of orthologs. *BMC Bioinformatics*.

[13] Minoda A., Weber A. P. M., Tanaka K., Miyagishima S.-Y. (2010). Nucleus-independent control of the rubisco operon by the plastid-encoded transcription factor Ycf30 in the red alga *Cyanidioschyzon merolae*. *Plant Physiology*.

[14] Cortese M. S., Caplan A. B., Crawford R. L. (2002). Structural, functional, and evolutionary analysis of *moeZ*, a gene encoding an enzyme required for the synthesis of the *Pseudomonas* metabolite, pyridine-2,6-bis(thiocarboxylic acid). *BMC Evolutionary Biology*.

[15] Collén J., Porcel B., Carré W. (2013). Genome structure and metabolic features in the red seaweed *Chondrus crispus* shed light on evolution of the Archaeplastida. *Proceedings of the National Academy of Sciences of the United States of America*.

[16] Depriest M. S., Bhattacharya D., López-Bautista J. M. (2013). The plastid genome of the red macroalga *Grateloupia taiwanensis* (Halymeniaceae). *PLoS ONE*.

[17] Glöckner G., Rosenthal A., Valentin K. (2000). The structure and gene repertoire of an ancient red algal plastid genome. *Journal of Molecular Evolution*.

[18] Hagopian J. C., Reis M., Kitajima J. P., Bhattacharya D., De Oliveira M. C. (2004). Comparative analysis of the complete plastid genome sequence of the red alga *Gracilaria tenuistipitata* var. liui provides insights into the evolution of rhodoplasts and their relationship to other plastids. *Journal of Molecular Evolution*.

[19] Janouškovec J., Liu S.-L., Martone P. T. (2013). Evolution of red algal plastid genomes: ancient architectures, introns, horizontal gene transfer, and taxonomic utility of plastid markers. *PLoS ONE*.

[20] Ohta N., Matsuzaki M., Misumi O. (2003). Complete sequence and analysis of the plastid genome of the unicellular red alga *Cyanidioschyzon merolae*. *DNA Research*.

[21] Reith M. E., Munholland J. (1995). Complete nucleotide sequence of the *Porphyra purpurea* chloroplast genome. *Plant Molecular Biology Reporter*.

[22] Wang L., Mao Y., Kong F. (2013). Complete sequence and analysis of plastid genomes of two economically important red algae: *Pyropia haitanensis* and *Pyropia yezoensis*. *PLoS ONE*.

[23] Campbell M. A., Presting G., Bennett M. S., Sherwood A. R. (2014). Highly conserved organellar genomes in the Gracilariales as inferred using new data from the Hawaiian invasive alga *Gracilaria salicornia* (Rhodophyta). *Phycologia*.

[24] Lyubetsky V. A., Seliverstov A. V., Zverkov O. A. (2013). Transcription regulation of plastid genes involved in sulfate transport in viridiplantae. *BioMed Research International*.

[25] Zverkov O. A., Rusin L. Y., Seliverstov A. V., Lyubetsky V. A. (2013). Study of direct repeats in micro evolution of plant mitochondria and plastids based on protein clustering. *Moscow University Biological Sciences Bulletin*.

[26] Zverkov O. A., Seliverstov A. V., Lyubetsky V. A. (2012). Plastid-encoded protein families specific for narrow taxonomic groups of algae and protozoa. *Molecular Biology*.

[27] Starkenburg S. R., Kwon K. J., Jha R. K. (2014). A pangenomic analysis of the Nannochloropsis organellar genomes reveals novel genetic variations in key metabolic genes. *BMC Genomics*.

[28] Benson D. A., Cavanaugh M., Clark K. (2013). GenBank. *Nucleic Acids Research*.

[29] Punta M., Coggill P. C., Eberhardt R. Y. (2012). The Pfam protein families database. *Nucleic Acids Research*.

[30] Sigrist C. J. A., de Castro E., Cerutti L. (2013). New and continuing developments at PROSITE. *Nucleic Acids Research*.

[31] Lyubetsky V. A., Rubanov L. I., Seliverstov A. V. (2010). Lack of conservation of bacterial type promoters in plastids of Streptophyta. *Biology Direct*.

[32] Seliverstov A. V., Lysenko E. A., Lyubetsky V. A. (2009). Rapid evolution of promoters for the plastome gene *ndhF* in flowering plants. *Russian Journal of Plant Physiology*.

[33] Homann A., Link G. (2003). DNA-binding and transcription characteristics of three cloned sigma factors from mustard (*Sinapis alba* L.) suggest overlapping and distinct roles in plastid gene expression. *European Journal of Biochemistry*.

[34] Lysenko E. A. (2007). Plant sigma factors and their role in plastid transcription. *Plant Cell Reports*.

[35] Lyubetsky V. A., Seliverstov A. V. (2003). Some algorithms related to finite groups. *Information Processes*.

[36] Lyubetsky V. A., Seliverstov A. V. (2004). Note on cliques and alignments. *Information Processes*.

[37] Bailey T. L., Boden M., Buske F. A. (2009). MEME SUITE: tools for motif discovery and searching. *Nucleic Acids Research*.

[38] Lyubetsky V. A., Zverkov O. A., Rubanov L. I., Seliverstov A. V. (2011). Modeling RNA polymerase competition: the effect of *σ*-subunit knockout and heat shock on gene transcription level. *Biology Direct*.

[39] Lyubetsky V. A., Zverkov O. A., Pirogov S. A., Rubanov L. I., Seliverstov A. V. (2012). Modeling RNA polymerase interaction in mitochondria of chordates. *Biology Direct*.

[40] Vladimirov A. A. (2013). Non-crossing matchings. *Problems of Information Transmission*.

[41] Muro-Pastor M. I., Florencio F. J. (2003). Regulation of ammonium assimilation in cyanobacteria. *Plant Physiology and Biochemistry*.

[42] Lopatovskaya K. V., Seliverstov A. V., Lyubetsky V. A. (2011). NtcA and NtcB regulons in cyanobacteria and rhodophyta chloroplasts. *Molecular Biology*.

[43] Forchhammer K. (2008). PII signal transducers: novel functional and structural insights. *Trends in Microbiology*.

